# A translational study on the survival and molecular mechanism of PD-L1 expression in EGFR-mutant NSCLC treated with osimertinib

**DOI:** 10.1016/j.isci.2025.114175

**Published:** 2025-11-21

**Authors:** Shidong Xu, Yangqian Chen, Xing Zhang, Xuexue Zhou, Jiacheng Dai, Yuanze Sun, Jie Zou, Yahui Chen, Linrui Ma, Zhe Huang, Liang Zeng, Yongchang Zhang

**Affiliations:** 1 Department of Pathology and Pathophysiology, School of Basic Medical Science, Central South University, Changsha 410078, China; 2 Department of Immunology, School of Basic Medical Science, Central South University, Changsha 410078, China; 3 Department of Medical Oncology, Hunan Cancer Hospital and The Affiliated Cancer Hospital of Xiangya School of Medicine, Central South University, Changsha 410013, China; 4 Department of Medical Oncology, Lung Cancer and Gastrointestinal Unit, Hunan Cancer Hospital and The Affiliated Cancer Hospital of Xiangya School of Medicine, Central South University, Changsha 410013, China

**Keywords:** Oncology, Therapeutics

## Abstract

Highly expressed programmed death-ligand 1 (PD-L1) has been associated with poor clinical outcomes in patients with epidermal growth factor receptor (*EGFR*)-mutated non-small cell lung cancer (NSCLC) receiving EGFR-tyrosine kinase inhibitors (TKIs). However, the prognostic significance of PD-L1 expression in the context of first-line osimertinib treatment remains unclear. In this retrospective study, we analyzed 317 patients with *EGFR*-mutated stage III–IV lung adenocarcinoma treated with first-line osimertinib. Patients with high PD-L1 expression demonstrated significantly shorter progression-free survival and overall survival compared to those with low PD-L1 expression. Transcriptomic analysis revealed upregulation of interferon-gamma (IFN-γ) and interleukin (IL)-6/JAK/STAT3 pathways in high PD-L1 tumors. Flow cytometry identified an increased proportion of CD56^bright^ natural killer (NK) cells in patients with high PD-L1 expression. *In vitro* experiments further demonstrated that IFN-γ induces PD-L1 expression via STAT3 activation. These findings provide evidence that baseline PD-L1 expression may serve as a prognostic biomarker for patients with *EGFR*-mutated NSCLC receiving first-line osimertinib and implicate the CD56^bright^ NK cell/IFN-γ/STAT3 axis in the regulation of PD-L1 expression.

## Highlights

• PD-L1 TPS ≥50% predicts poor prognosis in *EGFR*-mutated NSCLC on osimertinib
• CD56bright NK cells are enriched in patients with high PD-L1 expression
• IFN-γ upregulates PD-L1 expression via STAT3 activation in *EGFR*-mutated NSCLC
• PD-L1 status may serve as a prognostic biomarker for osimertinib treatment outcomes

## Graphical abstract

**Figure:**
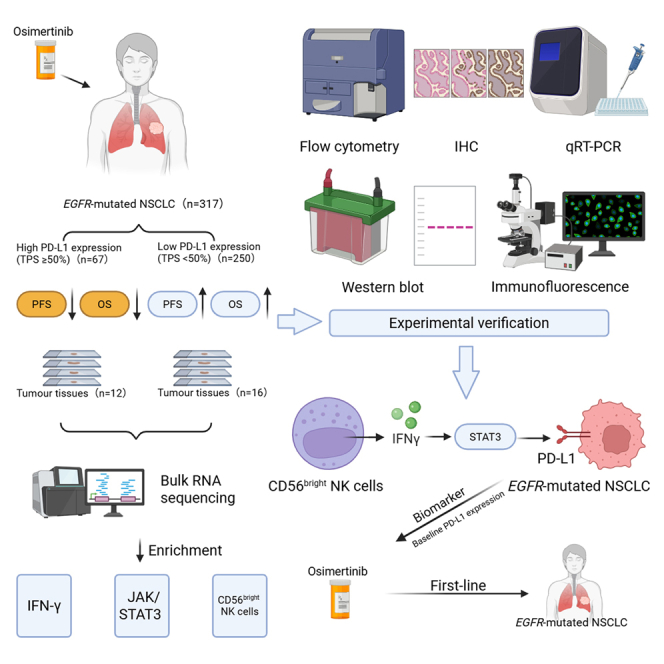

## Introduction

Non-small cell lung cancer (NSCLC) remains a leading cause of cancer-related mortality worldwide.^1^^,^^2^ For patients with advanced NSCLC who lack actionable genetic mutations, immune checkpoint inhibitors (ICIs) have become a cornerstone of first-line therapy.^3^ Among patients with driver mutations, those harboring epidermal growth factor receptor (EGFR) mutations represent the largest molecular subgroup, accounting for approximately 20%–30% of cases globally and up to 30%–50% in Asian populations.^4^ Tyrosine kinase inhibitors (TKIs) targeting EGFR have significantly improved clinical and survival outcomes in this population. Osimertinib, a third-generation EGFR-TKI, is currently approved for the treatment of NSCLC harboring EGFR-sensitizing mutations (exon 19 deletions or L858R) or the T790M resistance mutation.^5^

Programmed death-ligand 1 (PD-L1), a key immune checkpoint molecule, plays a central role in tumor immune evasion and is a validated therapeutic target in NSCLC.^6^ However, the prognostic significance of PD-L1 expression in *EGFR*-driven NSCLC remains controversial. While several studies have reported an association between high PD-L1 expression and poorer progression-free survival (PFS) following EGFR-TKI treatment in patients with *EGFR*-mutated lung adenocarcinoma,^7^ its relationship with overall survival (OS), particularly in those receiving first-line osimertinib, is yet to be clearly established.

Although activating *EGFR* mutations have been shown to induce PD-L1 expression,^8^^,^^9^ the proportion of patients with *EGFR*-mutated NSCLC who exhibit high PD-L1 expression remains relatively low. The biological mechanisms accounting for this heterogeneity in PD-L1 expression are not fully understood.

Several studies have explored the molecular pathways regulating PD-L1 expression in tumor cells. For instance, Garcia-Diaz et al. identified the interferon-gamma (IFN-γ)-JAK1/JAK2-STAT1/STAT2/STAT3-IRF1 signaling axis as a key regulator of PD-L1 expression in melanoma cells.^10^ Similarly, Zhang et al. demonstrated that activated EGFR can enhance PD-L1 expression in NSCLC cells via the IL-6/JAK/STAT3 pathway.^11^ These findings suggest that STAT3 signaling may be critically involved in modulating PD-L1 expression. IFN-γ is a well-established inducer of PD-L1.^12^ In humans, natural killer (NK) cells are broadly classified into two functional subsets: cytotoxic CD56^dim^CD16^+^ NK cells, which comprise approximately 90% of circulating NK cells, and cytokine-producing CD56^bright^ CD16^−^ NK cells, which secrete immunomodulatory factors such as IFN-γ.^13^ Based on the work of Chen et al. and Garcia-Diaz et al.,^10^^,^^14^ it is plausible that IFN-γ may activate STAT3 and drive PD-L1 expression in *EGFR*-mutated NSCLC, although this mechanism has yet to be elucidated in clinical specimens.

In this retrospective study, we assessed baseline PD-L1 tumor proportion score (TPS) in a large real-world cohort of patients with *EGFR*-mutated stage III–IV lung adenocarcinoma treated with first-line osimertinib. We investigated the association between baseline PD-L1 expression and survival outcomes, aiming to clarify its prognostic relevance. To further explore the molecular underpinnings of differential PD-L1 expression, we performed transcriptomic analysis, validated key findings using flow cytometry and immunohistochemistry, and conducted mechanistic studies in *EGFR*-mutated NSCLC cell lines to elucidate the role of the IFN-γ/STAT3 axis in regulating PD-L1 expression.

## Results

### Patient selection and baseline characteristics

**Figure 1:**
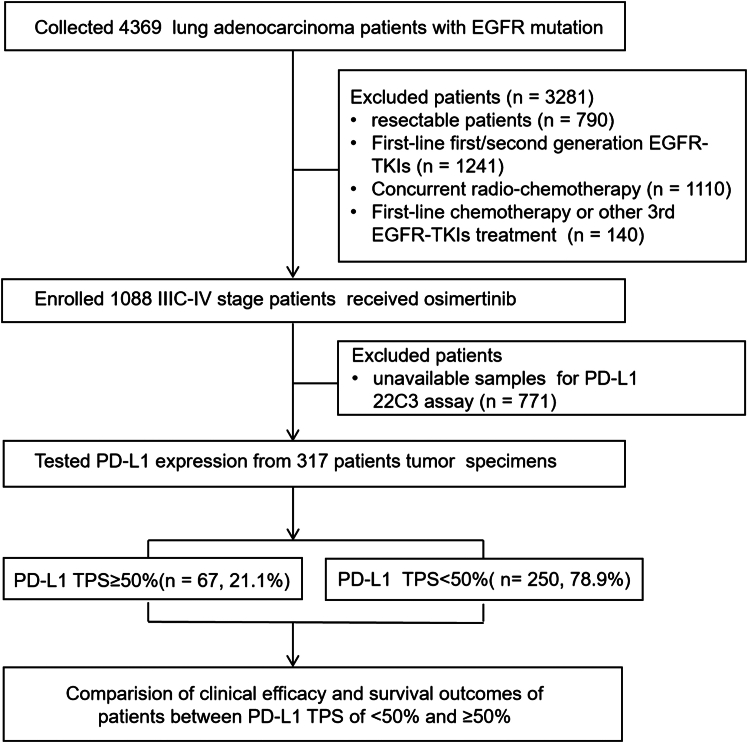
Patient screening Flow diagram illustrating the study design and patient flow.

A total of 4,369 patients diagnosed with *EGFR*-mutant lung adenocarcinoma at Hunan Cancer Hospital between November 2018 and November 2024 were screened for eligibility, as outlined in Figure 1. Among them, 317 patients met the inclusion criteria and were included in the final analysis. Based on a TPS cutoff of 50%, patients were stratified into two groups: low PD-L1 expression (TPS <50%) and high PD-L1 expression (TPS ≥50%). The low-expression group comprised 250 patients (78.9%), including 136 patients (42.9%) with TPS <1% and 114 patients (36.0%) with TPS 1%–49%. The remaining 67 patients (21.1%) were classified as having high PD-L1 expression (TPS ≥50%). Baseline clinical characteristics for all patients are summarized in Table 1. A comparative analysis between the high and low PD-L1 expression groups revealed no statistically significant differences in baseline clinicopathological features (Table 2).

**Table 1:** Baseline clinical characteristics

Characteristics	All patients (*N* = 317)
Age, median (range), years	59 (34, 91)
Sex, no. (%)	
Male	152 (48.0)
Female	165 (52.0)
Smoking history, no. (%)	
Smoker	111 (35.0)
Never smoked	206 (65.0)
ECOG PS, no. (%)	
0–1	294 (92.7)
2–3	23 (7.3)
Brain metastasis, no. (%)	
With	106 (33.4)
Without	211 (66.6)
Liver metastasis, no. (%)	
With	20 (6.3)
Without	297 (93.7)
Local treatment, no. (%)	
With	77 (24.3)
Without	240 (75.7)
*EGFR*-activating mutation type, no. (%)	
Exon 21 L858R	124 (39.1)
Exon 19 deletion	173 (54.6)
Others	20 (6.3)

ECOG PS: Eastern Cooperative Oncology Group Performance Status.

**Table 2:** Baseline clinical characteristics based on PD-L1 expression

Characteristics	PD-Ll expression		*p* value
	PD-L1<50%	PD-L1 ≥50%	
Age, median (range), years	64 (34, 83)	56 (36, 91)	–
Sex, no.(%)			0.286
Male	116 (46.4)	36 (53.7)	–
Female	134 (53.6)	31 (46.3)	–
Smoking history, no.(%)			0.894
Smoker	88 (35.2)	23 (34.3)	–
Never smoked	162 (64.8)	44 (65.7)	–
ECOG PS, no.(%)			0.546
0–1	233 (93.2)	61 (91.0)	–
2–3	17 (6.8)	6 (9.0)	–
Brain metastasis, no.(%)			0.115
With	89 (35.6)	17 (25.4)	–
Without	161 (64.4)	50 (74.6)	–
Liver metastasis, no.(%)			0.898
With	16 (6.4)	4 (6.0)	–
Without	234 (93.6)	63 (94.0)	–
Local treatment, no.(%)			0.882
With	61 (24.4)	16 (23.9)	–
Without	189 (75.6)	51 (76.1)	–
*EGFR*-activating mutation type, no.(%)			0.107
Exon 21 L858R	99 (39.6)	25 (37.3)	–
Exon 19 deletion	139 (55.6)	34 (50.7)	–
Others	12 (4.8)	8 (12.0)	–

ECOG PS: Eastern Cooperative Oncology Group Performance Status.

### High PD-L1 expression is associated with poor survival outcomes

We evaluated the clinical efficacy and survival outcomes following first-line osimertinib in 317 patients. The median PFS for the entire cohort was 17.4 months (95% confidence interval [95% CI]: 11.38–18.02 months) (Figure S1A), and the median OS was 37.5 months (95% CI: 31.38–43.52 months) (Figure S1B). The objective response rate (ORR) was 69.1% (219/317), and the disease control rate was 94.6% (300/317).

**Figure 2:**
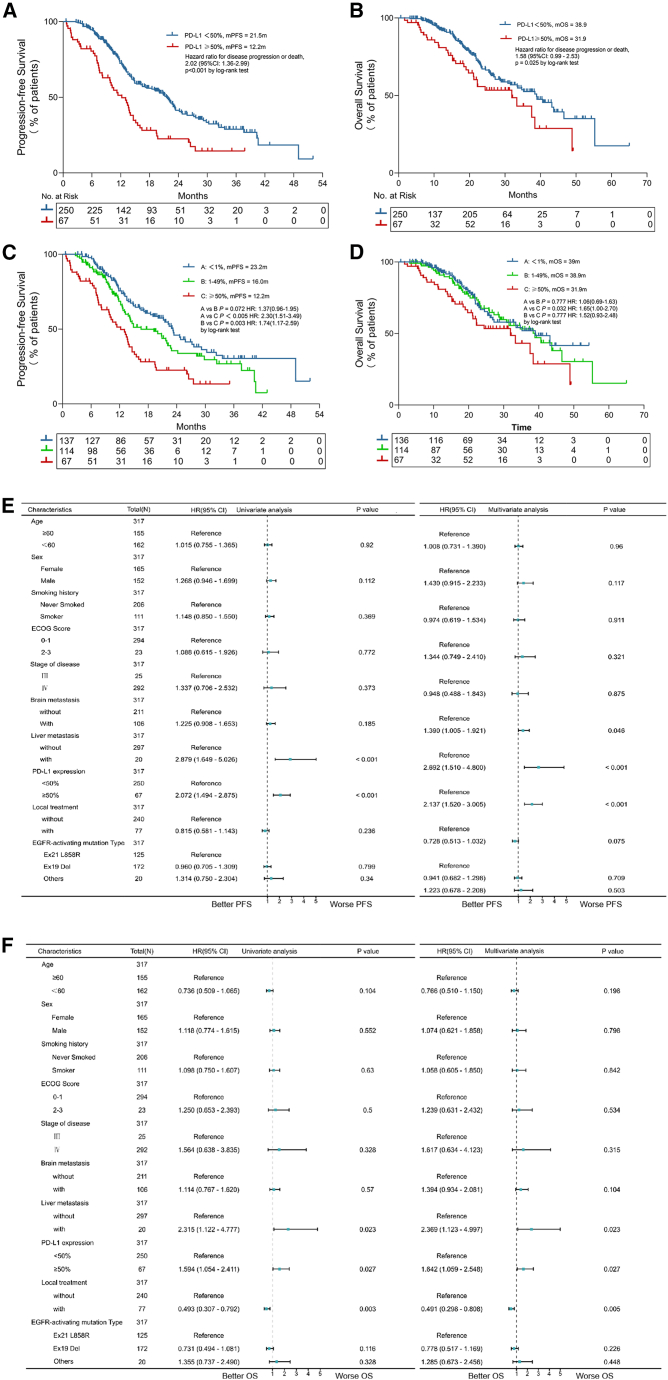
High PD-L1 expression is associated with poorer survival outcomes (A and B) Kaplan-Meier curve comparing progression-free survival (PFS) (A) and overall survival (OS) (B) with first-line osimertinib in patients with *EGFR*-mutated advanced NSCLC having high (tumor proportion score [TPS] ≥50%) or low (TPS <50%) PD-L1 TPS. (C and D) Outcomes of patients stratified by PD-L1 expression: PD-L1 negative (A, TPS <1%), PD-L1 low (B, TPS 1%–49%), and PD-L1 high (C, TPS ≥50%). Tick marks represent censored patients. Risk table below indicates the number of patient at risk at each time point. Hazard ratios and *p* values were calculated using the log rank test. (E and F) Univariate and multivariate Cox regression analyses identifying risk factors for PFS (E) and OS (F) with first-line osimertinib of patients with *EGFR*-mutated advanced NSCLC.

Subgroup analysis demonstrated no significant difference in ORR between high and low PD-L1 expression groups (70.1% vs. 68.8%, *p* = 0.712) (Table 3). However, median PFS was significantly shorter in the high PD-L1 expression group compared to the low PD-L1 group (12.2 vs. 21.5 months, *p* < 0.001) (Figure 2A). Similarly, OS was reduced in the high PD-L1 expression group (31.9 vs. 38.9 months, *p* = 0.025) (Figure 2B). Notably, no significant differences in PFS or OS were observed between patients with PD-L1 TPS <1% and those with TPS 1%–49% (Figures 2C and 2D).

**Table 3:** Objective response rate based on PD-Ll expression subgroups

	PD-L1 <50% (*n* = 250)	PD-L1 ≥50% (*n* = 67)	*p* value
Best response			0.712
Complete response	0 (0)	2 (3.0)	–
Partial response	172 (68.8)	45 (67.2)	–
Stable disease	72 (28.8)	9 (13.4)	–
Progressive disease	6 (2.4)	11 (16.4)	–

Univariate and multivariate analyses identified high baseline PD-L1 expression (*p* < 0.001) and liver metastases (*p* < 0.001) as independent predictors of shorter PFS (Figure 2E). For OS, high PD-L1 expression (*p* = 0.027), liver metastases (*p* = 0.023), and absence of local therapy (*p* = 0.003) were significant risk factors in univariate analysis and remained independently associated with worse OS in multivariate analysis (*p* = 0.027, *p* = 0.023, and *p* = 0.005, respectively) (Figure 2F).

### IFNG and IL-6/JAK/STAT3 signaling pathways are upregulated in patients with high PD-L1 expression

To explore the molecular basis of varying PD-L1 expression levels in *EGFR*-mutated NSCLC, baseline tumor tissues from 28 patients were subjected to bulk RNA sequencing. Of these, 12 patients had high PD-L1 expression (TPS ≥50%), and 16 patients had low PD-L1 expression (TPS <50%). Transcriptomic analysis revealed distinct gene expression profiles between the two groups (Figure S2A), suggesting underlying biological differences.

**Figure 3:**
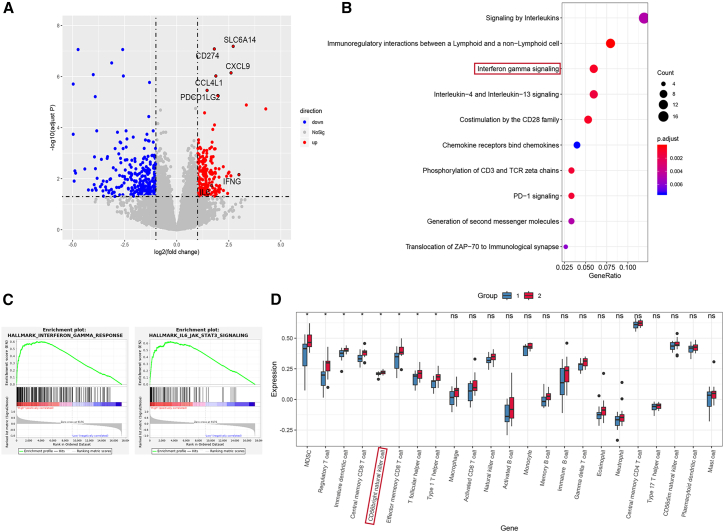
Upregulation of IFNG and IL-6/JAK/STAT3 signaling pathways in patients with high PD-L1 expression (A) Volcano plot showing differentially expressed genes between high and low PD-L1 expression groups. (B) Reactome pathway enrichment analysis of the differentially expressed genes in tumors with high PD-L1 expression compared to those with low expression. (C) Gene set enrichment analysis highlighting key pathways enriched in tumors with high PD-L1 expression compared to those with low expression. (D) Deconvolution analysis of immune cell infiltration between PD-L1 expression groups. Group 1: PD-L1 TPS <50%; group 2: PD-L1 TPS ≥50% (data are presented as mean ± SD; ∗*p* < 0.05; ns, not statistically significant).

Tumors with high PD-L1 expression exhibited elevated expression of *CD274* (PD-L1), *IFNG* (IFN-γ), and *IL-6* (Figure 3A). Pathway enrichment analysis indicated significant upregulation of the IFN-γ signaling and IL6-JAK-STAT3 signaling pathways in this group (Figures 3B and 3C).

Immune cell deconvolution analysis revealed significantly higher infiltration of immunosuppressive cells, including myeloid-derived suppressor cells and regulatory T cells (Figures 3D and S2C), in the high PD-L1 expression group. Among the differentially infiltrated immune populations, CD56^bright^ NK cells were significantly enriched in the high PD-L1 expression group (Figures 3D and S2B). Furthermore, a uniform manifold approximation and projection plot was constructed using external treatment-naive EGFR-mutant NSCLC single-cell sequencing data (Figure S2D).^15^ Analysis revealed that patients with high PD-L1 expression (P1) exhibited a higher proportion of CD56^bright^ NK cells compared to patients with low PD-L1 expression (P3, P4, P6, and P8) (Figures S2E and S2F). These findings linked the IFN-γ and JAK/STAT3 axes with PD-L1 expression and immune modulation.

### High PD-L1 expression is associated with elevated CD56^bright^ NK cells

To validate the association between PD-L1 expression levels and CD56^bright^ NK cells in *EGFR*-mutated NSCLC, we analyzed baseline peripheral blood samples from 10 patients with high PD-L1 expression and 16 patients with low PD-L1 expression. CD3^−^ T cells were isolated by flow cytometry, and surface markers CD56 and CD16 were analyzed.

**Figure 4:**
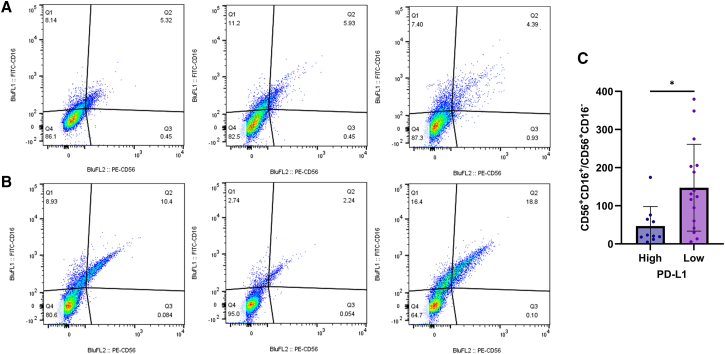
Increased proportion of CD56^bright^ NK cells in patients with high PD-L1 expression (A and B) Flow cytometric analysis of CD56^+^ and CD16^+^ cells within CD3^−^ populations in patients with high PD-L1 expression (A) and low PD-L1 expression (B). (C) Bar graphs showing the ratio of CD56^dim^ NK cell (CD56^+^ CD16^+^) subsets to CD56^bright^ NK cell (CD56^+^ CD16^−^) subsets across all patients (data are presented as mean ± SD; ∗*p* < 0.05).

Patients in the high PD-L1 group exhibited a significantly greater proportion of CD56^bright^ (CD56^+^CD16^−^) NK cells compared to the low PD-L1 expression group (Figures 4A and 4B). Additionally, the ratio of CD56^dim^ (CD56^+^CD16^+^) to CD56^bright^ NK cells was significantly reduced in the high PD-L1 expression group, confirming an enrichment of the immunoregulatory CD56^bright^ subset (Figure 4C).

### STAT3 expression is elevated in lung tumors with high PD-L1 expression

Given the transcriptomic findings, we next evaluated STAT3 protein levels via immunohistochemistry in baseline tumor tissues from 17 patients treated at other medical centers. Among these, 8 patients had high PD-L1 expression, and 9 patients had low PD-L1 expression.

**Figure 5:**
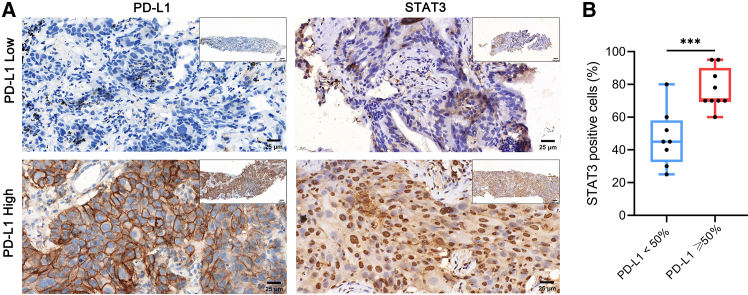
STAT3 expression is elevated in tumor tissues from patients with high PD-L1 expression (A) Representative immunohistochemical images showing PD-L1 and STAT3 expression in high vs. low PD-L1 tumors. Scale bars: 100 and 25 μm for full and zoom images. (B) Boxplots comparing the percentage of STAT3-positive cells in high vs. low PD-L1 expression groups (data are presented as mean ± SD; ∗∗∗*p* < 0.001).

STAT3 protein levels were significantly higher in tumor with high PD-L1 (Figure 5A), and the proportion of STAT3-positive cells was markedly elevated compared to the low PD-L1 group (Figure 5B), further supporting STAT3 involvement in the high PD-L1 tumors.

### IFN-γ induces PD-L1 expression via STAT3 activation in *EGFR*-mutated NSCLC cells

**Figure 6:**
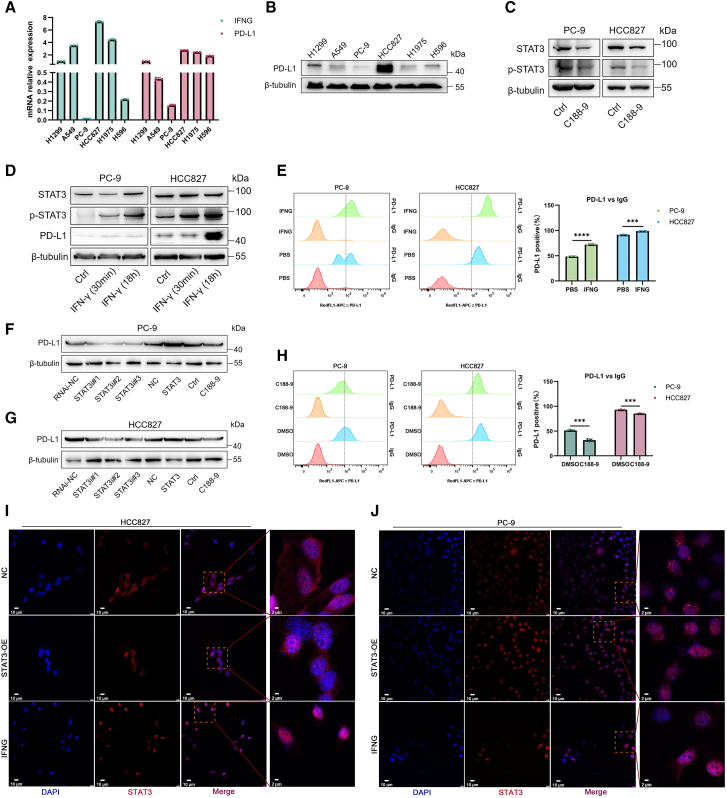
IFN-γ induces PD-L1 expression in *EGFR*-mutant NSCLC cell lines via STAT3 activation (A) Quantitative real-time PCR analysis of IFNG and PD-L1 gene expression in various lung cancer cell lines. (B–D) Western blot analysis demonstrating PD-L1 expression across cell lines (B), STAT3 phosphorylation following treatment with STAT3 inhibitor C188-9 in PC-9 (left) and HCC827 (right) cells (C), and PD-L1 expression and STAT3 phosphorylation after IFN-γ treatment for 30 min or 18 h in PC-9 (left) and HCC827 (right) cells. Ctrl denotes vehicle control. (E) Flow cytometry analysis of cell surface PD-L1 expression following IFN-γ stimulation (∗∗∗*p* < 0.001, ∗∗∗∗*p* < 0.0001). (F and G) Western blot analysis of PD-L1 expression in PC-9 (F) and HCC827 (G) cells after STAT3 knockdown, STAT3 overexpression, or C188-9 treatment. (H) Flow cytometry analysis of cell surface PD-L1 expression following C188-9 treatment (∗∗∗*p* < 0.001). (I and J) Immunofluorescence analysis of STAT3 localization in HCC827 (I) and PC-9 (J) cells that overexpress STAT3 (STAT3-OE) or treated with IFN-γ (IFNG). NC indicates negative control. Nuclei were stained with DAPI. Red boxes in the merge images highlight areas shown at higher magnification in the fourth column. Scale bars: 10 and 2 μm for full and zoom images (data are presented as mean ± SD).

We first examined the association between *CD274* and *IFNG* expression across various *EGFR*-mutated NSCLC cell lines. A positive association was observed, particularly in PC-9, H1975, HCC827, and H596 cells (Figure 6A). Based on endogenous PD-L1 expression levels, PC-9 (low PD-L1) and HCC827 (high PD-L1) cell lines were selected for further experiments (Figure 6B). STAT3 was overexpressed via plasmid transfection, and efficient knockdown was validated using three independent siRNAs and the STAT3 inhibitor C188-9 (Figures 6C, S2A, and S2B). IFN-γ treatment increased STAT3 phosphorylation and PD-L1 expression as demonstrated by western blotting (Figure 6D) and flow cytometry (Figure 6E).

Overexpression of STAT3 also upregulated PD-L1 expression, while STAT3 knockdown or C188-9 treatment with C188-9 reduced it (Figures 6F–6H). Immunofluorescence analysis revealed nuclear translocation of STAT3 following either STAT3 overexpression or IFN-γ stimulation (Figures 6I and 6J), indicating its transcriptional activation and role in PD-L1 induction.

Additionally, osimertinib was found to inhibit EGFR and STAT3 phosphorylation (Figures S4A and S4B), thereby reducing PD-L1 expression levels (Figure S4C). Modulation of PD-L1 expression through overexpression or knockdown (Figures S3C and S3D) significantly altered the cytotoxic effects of osimertinib: PD-L1 overexpression decreased tumor cell sensitivity to osimertinib, whereas PD-L1 knockdown markedly enhanced its cytotoxicity against tumor cells (Figures S4D–4F). These findings provide evidence for the suppressive effect of osimertinib on PD-L1 and suggest that its therapeutic efficacy is directly influenced by PD-L1 expression levels.

## Discussion

Osimertinib is the standard first-line treatment for patients with locally advanced or metastatic NSCLC harboring sensitizing *EGFR* mutations and T790M resistance mutations.^16^^,^^17^ While most patients with *EGFR*-mutated NSCLC experience durable responses to osimertinib, a subset develops early resistance, underscoring the need for predictive biomarkers to guide treatment strategies. Previous studies have suggested that EGFR pathway activation may promote immune evasion by upregulating PD-L1 expression, implicating PD-L1 as a potential resistance mechanism to EGFR-TKIs.^8^^,^^18^^,^^19^ Retrospective analyses have further shown that elevated baseline PD-L1 expression is associated with shorter PFS in patients with *EGFR*-mutated NSCLC.^20^^,^^21^ Liu et al. reported that high PD-L1 expression levels correlated with early resistance to first-generation EGFR-TKIs in a cohort of 186 patients,^20^ while Shiozawa et al. observed a similar association in 128 patients treated with osimertinib.^21^ However, these studies were limited to evaluating PFS and did not assess OS or explore the underlying biological mechanisms. In our study of 317 patients with *EGFR*-mutated lung adenocarcinoma, we demonstrated that high baseline PD-L1 expression (TPS ≥50%) was significantly associated with both shorter PFS and OS. These findings extend prior observations and support the role of PD-L1 expression as a prognostic biomarker in the setting of first-line EGFR-TKI therapy.

Interestingly, the proportion of patients with high PD-L1 expression in our cohort (21.2%) was notably higher than reported in previous studies (comparison of our study with others: *N* = 317 vs. 186 vs. 128, 21.1% vs. 12% vs. 5%, respectively).^20^^,^^21^ This discrepancy may reflect differences in sample size, population characteristics, or PD-L1 detection methods. Our study, which represents the largest cohort to date using the 22C3 assay, provides a more robust estimate of the prevalence of high baseline PD-L1 expression in *EGFR*-mutated lung adenocarcinoma within real-world clinical practice.

The median PFS and OS in our cohort were 17.4 months (95% CI: 11.38–18.02 months) and 37.5 months (95% CI: 31.38–43.52 months), respectively. These survival outcomes were slightly shorter than those reported in the osimertinib arm of the FLAURA study,^5^^,^^22^ which may be attributable to a higher proportion of patients with high PD-L1 expression or ethnic differences in our cohort.^23^ Notably, patients with high baseline PD-L1 expression in our cohort had significantly shorter PFS (12.2 vs. 21.5 months, *p* < 0.001) and OS (31.9 vs. 38.9 months, *p* = 0.025) than those with low expression. Multivariate analyses confirmed that high PD-L1 expression and liver metastases at baseline were independent adverse prognostic factors for both PFS and OS. Interestingly, baseline brain metastases were not associated with inferior outcomes, likely due to osimertinib’s robust intracranial activity.^5^^,^^24^^,^^25^

Although high PD-L1 expression predicts clinical benefit from ICIs in NSCLC,^26^^,^^27^ their predictive performance in patients with *EGFR*-mutated NSCLC remains limited. Several studies have shown that *EGFR*-mutated NSCLC is generally less responsive to ICIs,^28^^,^^29^^,^^30^ potentially due to the immunosuppressive or weakly immunogenic tumor microenvironment.^31^^,^^32^ However, recent data suggest that ICIs may become more effective following the development of resistance to EGFR-TKIs.^33^^,^^34^ These findings raise the possibility that patients with *EGFR*-mutated NSCLC with PD-L1 TPS ≥50% may benefit from ICI-based combination therapies, particularly those designed to alter the immune milieu.

**Figure 7:**
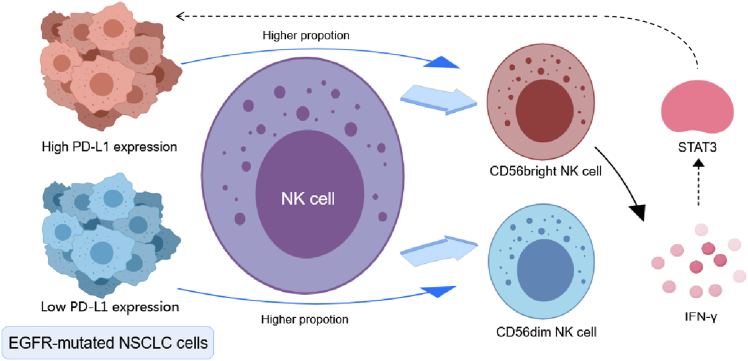
Schematic model of PD-L1 regulation in *EGFR*-mutated NSCLC cells *EGFR*-mutated NSCLC tumors with low PD-L1 expression harbor a higher proportion of CD56^dim^ natural killer (NK) cells, whereas tumors with high PD-L1 expression exhibit increased CD56^bright^ NK cells, which secrete IFN-γ, leading to STAT3 activation and upregulation of PD-L1 expression.

PD-L1 plays a central role in immune evasion by inhibiting effector T cell activity within the tumor microenvironment.^6^^,^^35^^,^^36^ In *EGFR*-mutated NSCLC, PD-L1 expression is associated with distinct patterns of immune infiltration.^37^^,^^38^^,^^39^ Multiple oncogenic and inflammatory pathways, including EGFR,^8^ ALK,^40^ mitogen-activated protein kinase,^41^ and cytokine signaling cascades, have been implicated in PD-L1 upregulation. In our study, high PD-L1 expression was associated with an increased proportion of CD56^bright^ NK cells, which secrete high levels of IFN-γ. This cytokine, in turn, activates the STAT3 signaling pathway, promoting PD-L1 expression. Although previous studies have demonstrated that the IFN-γ-STAT3-IRF1 signaling axis upregulates PD-L1 expression in malignant melanoma,^10^ these findings were primarily based on *in vitro* cell line models. In contrast, this study, utilizing real-world patient tissue and blood samples, provides the evidence that patients with EGFR-mutant NSCLC exhibiting high PD-L1 expression also display a higher proportion of CD56^bright^ NK cells for the first time. Furthermore, beyond assessing changes in STAT3 phosphorylation levels, we employed immunofluorescence to observe increased nuclear localization of STAT3 upon IFN-γ stimulation, indicating its activation and offering more direct evidence of IFN-γ-mediated regulation of STAT3. Finally, this study demonstrates, through analysis of real-world clinical samples, the upregulation of the IFNG and STAT3 pathways, elucidating the potential of PD-L1 as a biomarker for patients with EGFR-mutant NSCLC undergoing first-line osimertinib therapy (Figure 7). The high level of evidence presented underscores its translational clinical significance.

In conclusion, our study provides real-world clinical evidence that high baseline PD-L1 expression is an adverse prognostic factor in patients with advanced *EGFR*-mutated lung adenocarcinoma receiving first-line osimertinib therapy. Our findings support the utility of PD-L1 as a prognostic biomarker and highlight the need for risk stratification for this population. Furthermore, mechanistic data from transcriptomic and functional analyses suggest that CD56^bright^ NK cells and activation of the IFN-γ/IL-6/STAT3 axis contribute to elevated PD-L1 expression.

### Limitations of the study

This study primarily employed an *in vitro* model, wherein exogenous IFN-γ was added to investigate STAT3-mediated PD-L1 expression. Such an approach has inherent limitations in reflecting the complexity of the tumor immune microenvironment: firstly, endogenous IFN-γ is predominantly produced by immune cells such as CD56^bright^ NK cells and T lymphocytes, whose spatial distribution and concentration gradients differ significantly from the uniform exogenous IFN-γ applied *in vitro*.^42^^,^^43^ Secondly, the *in vitro* model cannot simulate the intricate interactions among diverse cell types—including immune cells, stromal cells, and tumor-associated fibroblasts—that can modulate downstream signaling pathways through cytokine networks.^42^^,^^43^ Lastly, it is challenging to replicate the tumor microenvironmental conditions such as hypoxia and acidity.^44^^,^^45^ Collectively, these limitations imply that *in vitro* experiments are insufficient to comprehensively capture the dynamic production of IFN-γ by CD56^bright^ NK cells *in vivo*, or to fully elucidate the influence of endogenous IFN-γ on PD-L1 expression and subsequent clinical outcomes. Therefore, investigations should incorporate multidimensional microenvironmental factors for validation in the future, enabling a more accurate assessment of the role of endogenous IFN-γ in tumor immune regulation and clinical prognosis. These insights deepen our understanding of the immunobiology underlying resistance to EGFR-TKIs and may inform the development of novel therapeutic strategies combining targeted therapy and immunomodulation.

## Resource availability

### Lead contact

Further information and requests for resources should be directed to and will be fulfilled by the lead contact, Yongchang Zhang (zhangyongchang@csu.edu.cn).

### Materials availability

This study did not generate new unique reagents.

### Data and code availability

• The RNA-sequencing data reported in this article have been deposited at GSA-Human (Genome Sequence Archive in BIG Data Center, Beijing Institute of Genomics, Chinese Academy of Sciences, https://bigd.big.ac.cn/gsa-human), and the accession number is provided in the key resources table. In compliance with national regulations regarding sharing of human genetic resources, the RNA sequencing data must be managed under controlled access. Requests to access data should follow the GSA’s “Data Access Request Guidance,” available at https://ngdc.cncb.ac.cn/gsa-human/document. Applicants will be required to complete and sign a data access agreement. The data access committee, guided by the DAC chair (Yongchang Zhang, zhangyongchang@csu.edu.cn), regulates access in accordance with institutional and national guidelines. Data are to be used solely for research purposes as approved in the data access agreement.
• This article does not report original code.
• Any additional information required to reanalyze the data reported in this article is available from the lead contact upon request.

## Acknowledgments

The authors are grateful to Prof. Xiangfan Liu and Ning Chen from Rui Jin Hospital Shanghai Jiaotong University School of Medical for their generous donation of H1299 and A549 cell lines. The authors would also like to thank Dr. Analyn Lizaso for her editing assistance. This work received financial support from the 10.13039/501100001809National Natural Science Foundation of China (grant nos. 82222048 and 82173338).

## Author contributions

S.X., Yangqian Chen, and X. Zhang: conceptualization, data curation, formal analysis, investigation, methodology, writing – original draft, and writing – review and editing. X. Zhou and J.D.: formal analysis, software, methodology, and writing – review and editing. Y.S., J.Z., and Yahui Chen: validation, investigation, visualization, and writing – review and editing. L.M. and Z.H.: conceptualization, investigation, and methodology. L.Z. and Y.Z.: conceptualization, resources, formal analysis, supervision, funding acquisition, validation, writing – original draft, and writing – review and editing.

## Declaration of interests

The authors declare no financial interests.

## STAR★Methods

### Key resources table

**Table:** 

REAGENT or RESOURCE	SOURCE	IDENTIFIER
**Antibodies**		

PD-L1	Cell Signaling Technology	Cat# 13684; RRID: AB_2687655
STAT3	Cell Signaling Technology	Cat# 9139; RRID: AB_331757
Phospho-STAT3 (Tyr705)	Cell Signaling Technology	Cat# 9145
EGFR	Cell Signaling Technology	Cat# 4267
Phospho-EGFR (Tyr1068)	Cell Signaling Technology	Cat# 3777
β-Tubulin	Cell Signaling Technology	Cat# 2146
APC anti-human CD3	Biolegend	Cat# 981012; RRID: AB_2876776
PE anti-human CD56	Biolegend	Cat# 985902; RRID: AB_2910522
FITC anti-human CD16	Biolegend	Cat# 980112; RRID: AB_2876771
APC anti-human PD-L1	Proteintech	Cat# APC-FcA98062; RRID: AB_3672209
Fluorescent secondary antibody	Thermo Fisher Scientific	Cat# a32794; RRID: AB_2762834
DAPI	Thermo Fisher Scientific	Cat# D1306

**Chemicals, peptides, and recombinant protein**		

C188-9	Selleckchem	Cat# S8605
Osimertinib	Selleckchem	Cat# S7292
Recombinat Human IFN-γ protein	Proteintech	Cat# HZ-1301
RPMI-1640 medium	Gibco	Cat# 11875119
Fetal Bovine Serum (FBS)	ZQXZBIO	Cat# ZQ500-S
0.25% Trypsin-EDTA	Gibco	Cat# 25200056
Penicillin and Streptomycin	Gibco	Cat# 15140122
Dimethyl sulfoxide	MedChemExpress	Cat# HY-Y0320C
Crystal Violet	MedChemExpress	Cat# HY-B0324A

**Critical commercial assays**		

Lipofectamine 3000 Transfection	Thermo Fisher Scientific	Cat# L3000008
Pierce™ BCA Protein Assay Kit	Thermo Fisher Scientific	Cat# 23225
RIPA buffer	Thermo Fisher Scientific	Cat# 89900
RNA Extraction Kit	Accurate Biology	Cat# AG21024
M-MLV RT-PCR Kit	Accurate Biology	Cat# AG11604
SYBR Green PCR Master Mix	Accurate Biology	Cat# AG11762
Cell Counting Kit-8	MedChemExpress	Cat# HY-K0301

**Experimental models: cell lines**		

PC-9	Chinese Academy of Sciences	Cat# SCSP-5085
HCC827	Chinese Academy of Sciences	Cat# SCSP-538
H1975	Chinese Academy of Sciences	Cat# SCSP-597
H596	ZQXZBIO	Cat# ZQ0013
H1299	Provided by Prof. Xiangfan Liu, Ruijin Hospital Shanghai Jiaotong University School of Medical	N/A
A549	Provided by Prof. Ning Chen, Ruijin Hospital Shanghai Jiaotong University School of Medical	N/A

**Oligonucleotides**		

Primers for qPCR, see STAR Methods	This paper	N/A
siRNA and plasmid of STAT3 and PD-L1, see STAR Methods	This paper	N/A

**Deposited data**		

RNA-seq data	This paper (https://bigd.big.ac.cn/gsa-human)	HRA011985

**Software and algorithms**		

GraphPad Prism version 10	GraphPad Software	N/A
FlowJo v10.0.7	Leonard Herzenberg	http://www.flowjo.com/

### Experimental model and study participant details

#### Human tissue and blood samples

The non-small cell lung cancer tissues for RNA-seq and immunohistochemistry staining were derived from 28 (13 males and 15 females aged 48-64 years) and 17 (8 males and 9 females aged 44-59 years) East Asian Chinese *EGFR*-mutated NSCLC patients, respectively. The blood samples for the flow cytometry were obtained from 26 East Asian *EGFR*-mutated Chinese NSCLC patients (11 males and 15 females aged 52-75 years). The samples were randomly selected from treatment-naive patients who underwent PD-L1 testing at Hunan Cancer Hospital between 2022 and 2024. Similar findings are reported for both sexes in the study. All patients were informed and provided written informed consent for the use of their specimens prior to the liquid or tissue biopsy. The acquisition of human tissue and blood samples was approved by the Hunan Cancer Hospital Ethics Committee with patient consent (Ethical approval number: KY2025604). The study protocol was approved by the Institutional Review Board (IRB) of Hunan Cancer Hospital.

#### Cell lines

Human NSCLC cell lines (PC-9, HCC827 and H1975) were obtained from the Chinese Academy of Sciences cell bank (Shanghai, China). NSCLC cell line H596 was purchased from ZQXZBIO. H1299 and A549 cell lines were kindly provided by Prof. Xiangfan Liu and Ning Chen (Rui Jin Hospital Shanghai Jiaotong University School of Medicine). Cells were cultured in Roswell Park Memorial Institute medium 1640 (RPMI-1640) medium supplemented with 10% fetal bovine serum (FBS), 50 μg/mL penicillin, and 50 μg/mL streptomycin, and maintained at 37°C in a 5% CO2 humidified incubator. The cell lines were authenticated by STR profiling and tested routinely before use to avoid mycoplasma contamination.

### Method details

#### Patients screening

This retrospective study screened patients diagnosed with stage IIIB-IV NSCLC at our center between November 1, 2018, and November 1, 2024. Inclusion criteria were as follows: (1) confirmed EGFR-sensitizing mutations (exon 19 deletion or L858R) identified via next-generation sequencing (NGS); (2) pre-treatment PD-L1 expression assessed using the 22C3 antibody; (3) first-line treatment with osimertinib; and (4) presence of at least one measurable lesion. Patients who received osimertinib in combination with chemotherapy or anti-angiogenic agents as first-line therapy were excluded.

#### Clinical outcomes & assessments

All patients received osimertinib at a daily oral dose of 80 mg. Tumor assessments were conducted at baseline, every 6 weeks (±1 week) for the first 18 months, and subsequently every 12 weeks (±1 week) until disease progression. Imaging assessments included contrast-enhanced computed tomography (CT) scans of the chest and abdomen, and magnetic resonance imaging (MRI) of the brain.

Primary outcomes were progression-free survival (PFS) and overall survival (OS), PFS was defined as the time from osimertinib initiation to disease progression or death from any cause. OS was defined as the time from osimertinib initiation to death from any cause or last follow-up. Secondary outcomes included objective response rate (ORR) and disease control rate (DCR), ORR was defined as the proportion of patients with a confirmed response status of complete response (CR) or partial response (PR). DCR was defined as the proportion of patients who achieved a best overall response of CR, PR, or stable disease (SD). Treatment responses were assessed using Response Evaluation Criteria in Solid Tumor (RECIST) version 1.1.

#### Bulk RNA sequencing and bioinformatics analysis

A total of 28 tumor biopsy specimens from patients with *EGFR*-mutated NSCLC were collected. RNA was extracted and libraries were prepared using the TruSeq standard mRNA library preparation kit (Illumina). Library quality was assessed using the Agilent 2100 Bioanalyzer and quantitative real-time polymerase chain reaction (qRT-PCR), followed by sequencing on the Illumina HiSeq 4000 platform. Quality control of raw sequencing reads was performed using FastQC and Trimmomatic. Transcript alignment and quantification were conducted using TopHat2. Differential gene expression analysis was performed using the DESeq2 package, and p-values were adjusted using the Benjamini-Hochberg (BH) method. Functional enrichment and pathway analyses were conducted using the ClusterProfiler package, while immune cell infiltration was analyzed using the GSVA package.

#### Processing of single-cell RNA sequencing (scRNA-seq) data

The scRNA-seq dataset is registered under GEO accession number GSE171145, comprising five lung adenocarcinoma (LUAD) samples with EGFR mutations and four samples with wild-type EGFR.^15^ The preprocessing workflow was conducted in accordance with previously published protocols. For cell type identification, classification was performed using a resolution of 0.8 and characteristic cell type-specific marker genes. Subsequently, further clustering analysis was conducted on NK cells from five NSCLC patients harboring EGFR mutations (P1, P3, P4, P6, P8), with the aim of delineating CD56^bright^ and CD16^bright^ NK cell subtypes (PD-L1 TPS of P1 = 65%, PD-L1 TPS of P3, P4, P6, and P8< 1%). The number and proportion of each subtype within individual patients were quantified, and data visualization was performed utilizing the “ggplot” package.

#### Reagents and antibodies

C188-9 (S8605) and osimertinib (S7292) were purchased from Selleck (Houston, TX, USA). Recombinant human IFN-γ protein (HZ-1301) was obtained from Proteintech (Rosemont, IL, USA). Primary antibodies against PD-L1 (#13684), STAT3 (#9139), p-STAT3 (#9145), EGFR (#4267), p-EGFR (#3777), and β-Tubulin (#2146) were purchased from Cell Signaling Technology (Danvers, MA, USA). PE anti-human CD56 (985902) and FITC anti-human CD16 (980112) antibodies were obtained from BioLegend (San Diego, CA, USA). APC anti-human PD-L1 antibody was purchased from Proteintech. Alexa Fluor-conjugated secondary antibody (a32794) was purchased from Thermo Fisher Scientific (Waltham, MA, USA).

#### STAT3 and PD-L1 knockdown and overexpression

STAT3 and PD-L1 knockdown was achieved using siRNAs targeting STAT3/PD-L1 mRNA. STAT3 and PD-L1 overexpression was achieved via plasmid transfection containing the full-length STAT3/PD-L1 coding sequence under a constitutive promoter. All siRNAs and plasmids were purchased from OBiO Technology (Shanghai, China) and validated by sequencing. Transfections were performed using Lipofectamine 3000 (Thermo Fisher Scientific) following the manufacturer’s instructions.

#### Flow cytometry

Single-cell suspensions from peripheral blood (post-red blood cell lysis) or tumor tissues were incubated with fluorescently labeled monoclonal antibodies at 4°C for 30 minutes. Samples were analyzed using a FACSAria III cell sorter (BD Biosciences) and a DxP Athena flow cytometer (Cytek). Data analysis was performed using FlowJo software (Ashland, OR, USA).

#### Immunohistochemistry (IHC)

PD-L1 expression in baseline tissue samples from Hunan Cancer Hospital was assessed using the PD-L1 Clone 22C3 assay kit (pharmDx). Tumor samples were fixed, paraffin-embedded, sectioned (4 μm), and stained with primary antibodies overnight at 4°C. The tumor proportion score (TPS) was calculated as the percentage of tumor cells exhibiting membranous staining among ≥100 viable cells, and categorized into TPS <50% and TPS ≥50%. For 17 cases from the center, PD-L1 and STAT3 IHC staining was performed, and the percentage of positive cells was quantified.

#### qRT-PCR analysis

Total RNA was extracted using the RNA Extraction Kit (AG, #AG21024, China), followed by reverse transcription with Super M-MLV reverse transcriptase (AG). SYBR Green PCR Master Mix (AG) was used for qRT-PCR on a CFX Opus 96 real-time system (Bio-Rad). Primer sequences are as follows:

PD-L1, sense: 5′-TGCCGACTACAAGCGAATTACTG-3′; anti-sense: 5′-CTGCTTGTCCAGATGACTTCGG-3′.

IFNG, sense: 5′-GAGTGTGGAGACCATCAAGGAAG-3′; anti-sense: 5′- TGCTTTGCGTTGGACATTCAAGTC-3′.

ACTB, sense: 5′-CACCATTGGCAATGAGCGGTTC-3′; anti-sense: 5′- AGGTCTTTGCGGATGTCCACGT-3′.

Gene expression levels were calculated using the 2ˆ-ΔΔCt method.

#### Western blotting (WB)

Cells were lysed using RIPA buffer (Thermo Fisher Scientific, #89900) and protein concentration were determined by bicinchoninic acid (BCA) assay (Thermo Fisher Scientific, #23225). Equal amounts of protein (30 μg) were resolved via SDS-polyacrylamide gel (10%) electrophoresis, transferred to PVDF membranes (Millipore, IPVH00010), and blocked with 5% non-fat milk (Bio-Rad, #170-6404) for 1 hour at room temperature. Membranes were incubated with primary antibodies overnight at 4°C, followed by secondary antibody incubation, and signal detection using chemiluminescence.

#### Immunofluorescence (IF)

Cells grown on glass coverslips were transfected with STAT3 plasmids or treated with IFN-γ, then fixed in 4% paraformaldehyde for 15 min at room temperature. After blocking with BSA for 2 hours, cells were incubated with STAT3 primary antibody at 4°C overnight, followed by incubation with Alexa Fluor 568-conjugated secondary antibodies for 2 hours at room temperature. Nuclei were counterstained with DAPI. Images were captured using a fluorescence microscope with appropriate filters (Leica).

#### CCK-8 assay

Tumor cells were plated in a 96-well plate at a density of 5×10^3^ cells per well, grown overnight, and treated with osimertinib at the concentration of 10 nM for the CCK-8 assay. Control cells were treated with an equivalent amount of DMSO. Each well was added with 10 μL of CCK-8 solution, and the OD450 was evaluated with an enzyme labeling instrument after 2h incubation.

#### Colony formation assay

1×10^3^ cells cells were plated in six-well plates and cultured in complete medium. The cells were treated with 10 nM osimertinib agents and incubated at 37°C in 5% CO2 for 14 days. The colonies were fixed with 4% paraformaldehyde (PFA), stained with 0.1% crystal violet, imaged, and counted.

#### Statistical analysis

Categorical variables were compared using Fisher’s exact test or chi-square test. Kaplan-Meier curves with log-rank tests were used to evaluate PFS and OS. Variables with p < 0.05 in univariate analysis were included into multivariate Cox proportional hazards model. Experimental data were presented as mean ± standard deviation from at least three independent experiments. Statistical analyses were performed using GraphPad Prism version 10. One-way ANOVA or unpaired t-tests were used where appropriate. Significance was denoted using asterisks as : ∗p < 0.05, ∗∗p < 0.01, ∗∗∗p < 0.001, and ∗∗∗∗p < 0.0001.
