# Supplementary material for: A translational study on the survival and molecular mechanism of PD-L1 expression in EGFR-mutant NSCLC treated with osimertinib
Source: iScience. 2025 Nov 21;28(12):114175. doi: 10.1016/j.isci.2025.114175 (PMC12723275; doi:10.1016/j.isci.2025.114175)
Supplement: Document S1. Figures S1–S4 [file mmc1.pdf]

iScience, Volume *28*

Supplemental information

A translational study on the survival and molecular mechanism of PD-L1 expression in EGFR-mutant NSCLC treated with osimertinib

Shidong Xu, Yangqian Chen, Xing Zhang, Xuexue Zhou, Jiacheng Dai, Yuanze Sun, Jie Zou, Yahui Chen, Linrui Ma, Zhe Huang, Liang Zeng, and Yongchang Zhang

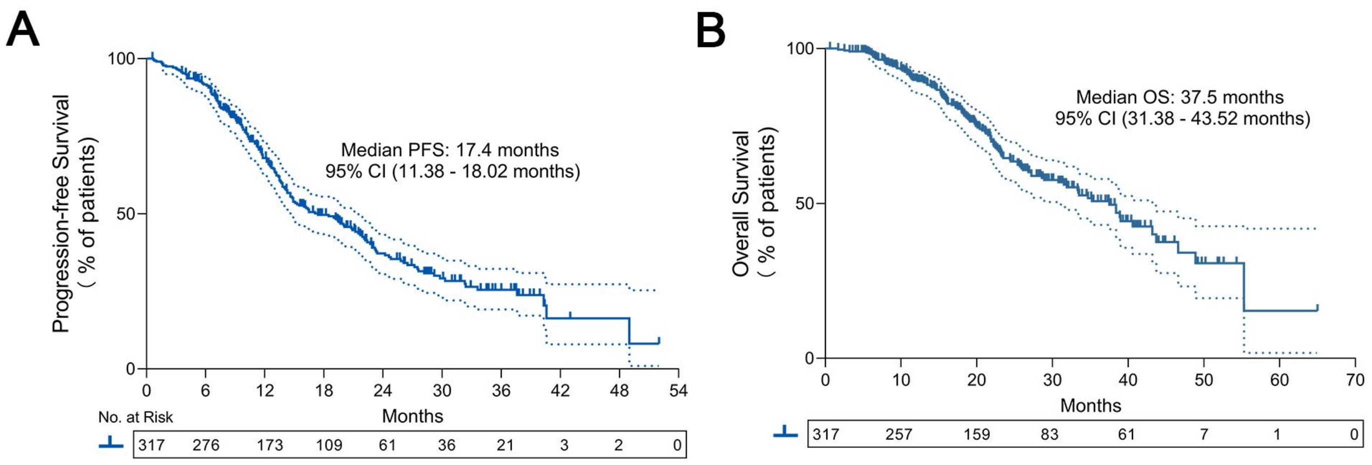

Figure S1. Kaplan-Meier survival curves depicting the progression-free survival (PFS; **A**) and overall survival (OS, **B**) in patients with *EGFR*-mutated advanced NSCLC treated with first-line osimertinib. Tick marks represent censored patients. Risk table below indicates the number of patient at risk at each time point. Hazard ratios and p-values were calculated using the log-rank test. Abbreviations: CI, confidence intervals; HR, hazard ratio; mOS, median overall survival; mPFS, median progression-free survival.

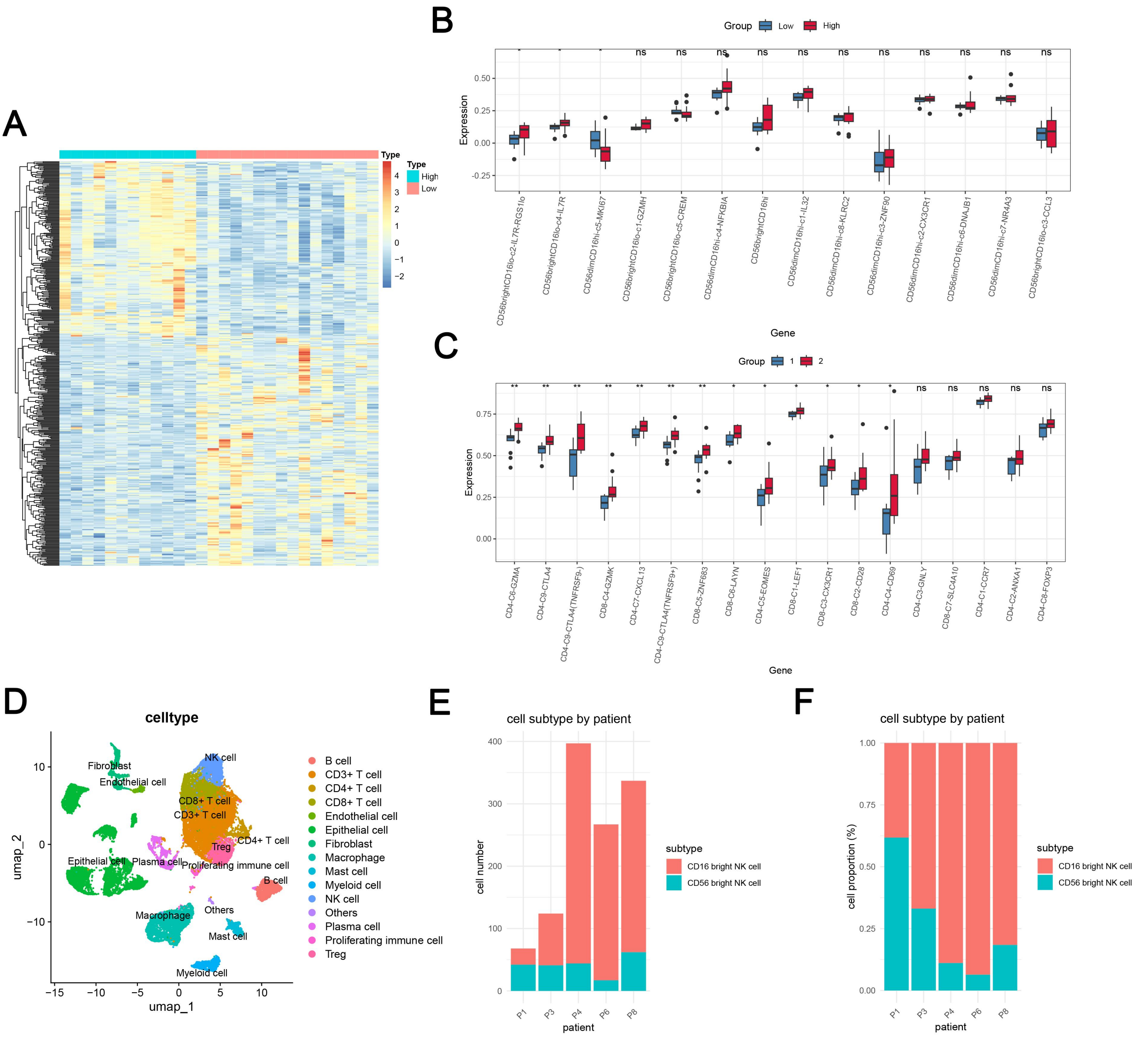

Figure S2. A. Heat map of differentially expressed gene clustering. **B**. Deconvolution analysis of natural killer cell subpopulations.
C. Deconvolution analysis of T cell subpopulations.
D. UMAP plot of the 40,799 total cells from treatment-naïve samples, color-coded by cell type.
E. The quantity of CD16 bright NK cells (above) and CD56 bright NK cells (bottom) in individual *EGFR*(+) NSCLC samples.
F. The proportion of CD16 bright NK cell subtype (above) and CD56 bright NK cell subtype (bottom) in individual *EGFR*(+) NSCLC samples. (Data are presented as mean ± SD; *p<0.05; **p<0.01; ns, not statistically significant).

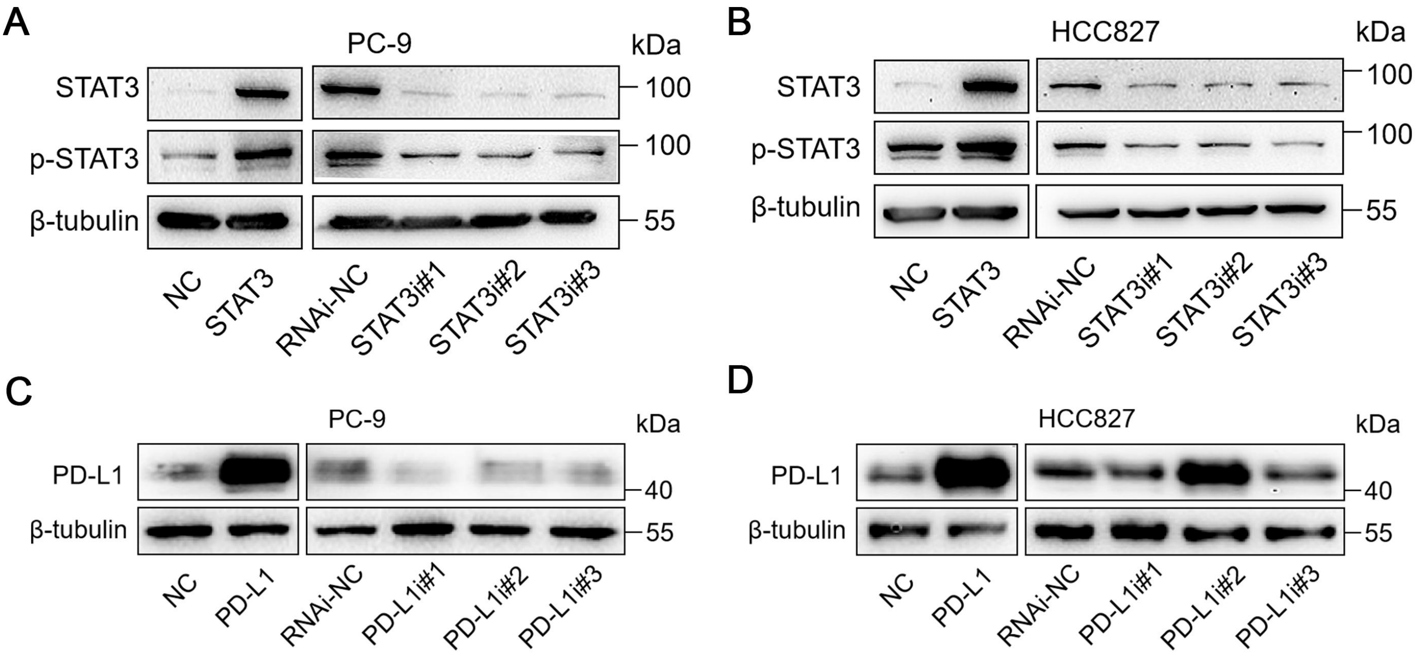

Figure S3. Western blot analysis demonstrated the expression levels of PD-L1 (**C**, **D**), total STAT3, and phosphorylated STAT3 (p-STAT3) (**A**, **B**) in PC-9 cells and HCC827 cells. The left panels depict overexpression of PD-L1 or STAT3, whereas the right panels illustrate knockdown of PD-L1 or STAT3 achieved via RNA interference. NC, negative control. β-tubulin was used as a loading control.

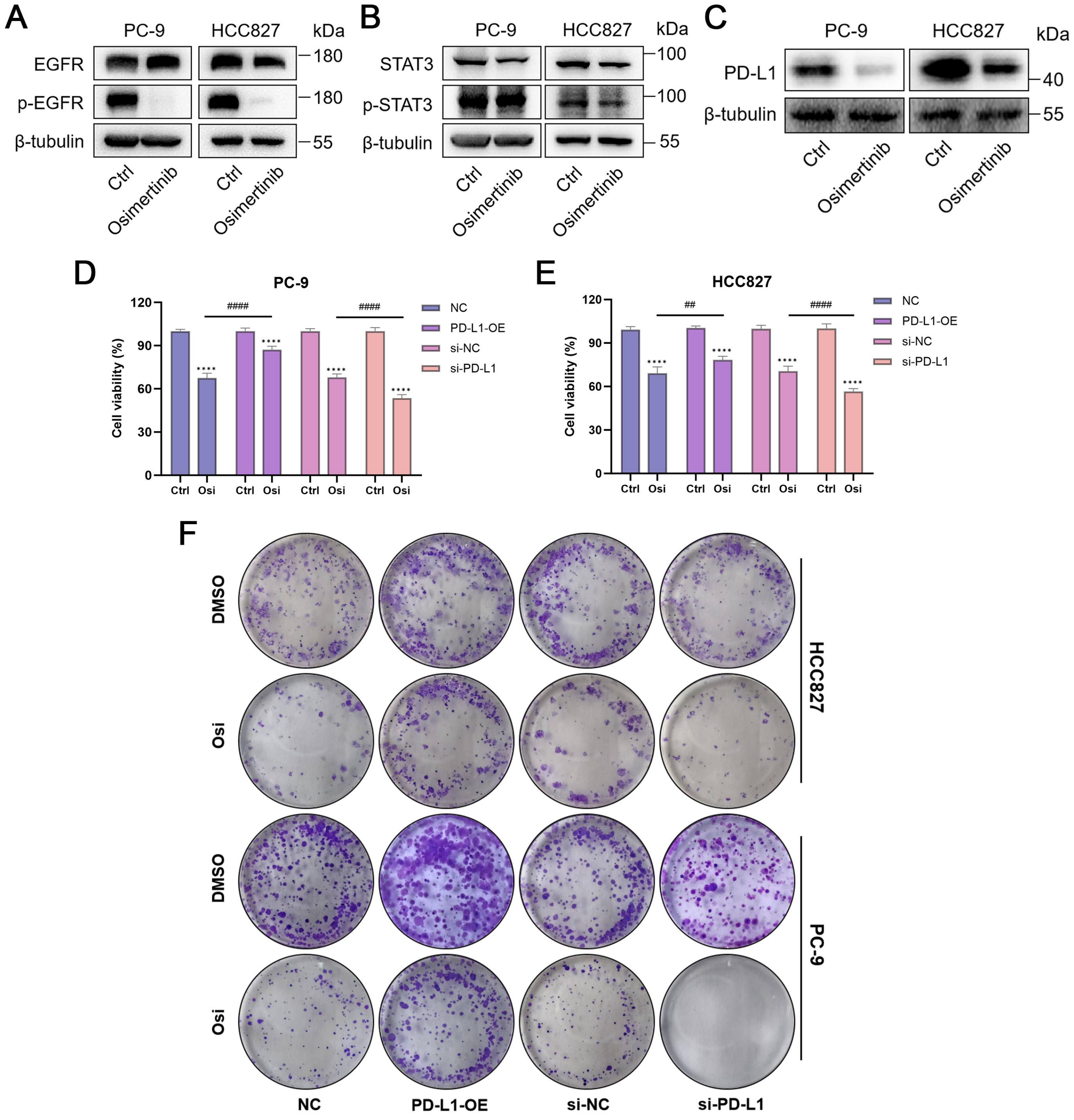

Figure S4. A-C. Western blot analysis of total EGFR and phosphorylated EGFR (p-EGFR) (**A**), total STAT3 and phosphorylated STAT3 (p-STAT3) (**B**) and PD-L1 (**C**) with osimertinib treatment in PC-9 cells (left panels) and HCC827 cells (right panels). β-tubulin was used as a loading control.
D-E. The CCK-8 assay demonstrated the cytotoxic effects of osimertinib treatment in PC-9 (**D**) and HCC827 (**E**) cell lines following either PD-L1 overexpression or knockdown.
F. Colony formation assay of cells treated with osimertinib following PD-L1 overexpression or knockdown. (Data are presented as mean ± SD; **p<0.01; ****p<0.0001;).
